# Seeking a deeper understanding of ‘distributed health literacy’: A systematic review

**DOI:** 10.1111/hex.13450

**Published:** 2022-02-18

**Authors:** Danielle M. Muscat, Danielle Gessler, Julie Ayre, Ole Norgaard, Iben R. Heuck, Stefanie Haar, Helle T. Maindal

**Affiliations:** ^1^ Sydney Health Literacy Lab, Sydney School of Public Health, Faculty of Medicine and Health The University of Sydney Sydney Australia; ^2^ School of Psychology, Faculty of Science The University of Sydney Sydney Australia; ^3^ Danish Diabetes Knowledge Center, Education, Copenhagen University Hospital Steno Diabetes Center Copenhagen Herlev Denmark; ^4^ Department of Public Health Aarhus University Aarhus Denmark; ^5^ Health Promotion Research, Copenhagen University Hospital Steno Diabetes Center Copenhagen Herlev Denmark

**Keywords:** distributed health literacy, health literacy, social context, social support, systematic review

## Abstract

**Background:**

Previous research suggests that it would be useful to view health literacy as a set of ‘distributed competencies’, which can be found dispersed through the individual's social network, rather than an exclusively individual attribute. However, to date there is no focused exploration of how distributed health literacy has been defined, conceptualized or assessed in the peer‐reviewed literature.

**Aims:**

This systematic review aimed to explore: (1) definitions and conceptual models of distributed health literacy that are available from the peer‐reviewed literature; and (2) how distributed health literacy has been measured in empirical research.

**Methods:**

We searched MEDLINE, Embase, CINAHL, PsycInfo, Scopus, ERIC and Web of Science using truncated versions of the keywords ‘literacy’ and ‘distributed’ (within five words' distance). We collated the definitions and conceptual models of distributed health literacy, and report on how health literacy has been measured in empirical research studies. Findings related to distributed health literacy from included manuscripts were synthesized using thematic synthesis.

**Results:**

Of the 642 studies screened, 10 were included in this systematic review. The majority were empirical manuscripts reporting on qualitative research in one of five countries, with two reviews, one conceptual analysis and one quantitative study. Edwards' definition of distributed health literacy, which emphasizes the health literacy abilities, skills and practices of others that contribute to an individual's level of health literacy was widely applied in a variety of clinical and geographical settings. However, we did not identify any quantitative instruments which directly measured distributed health literacy. There was significant variability in questions used to explore the concept qualitatively, and discrepancies across studies in regard to (a) what constitutes distributed health literacy and what does not (e.g., general social support), and (b) the relationship between distributed health literacy and other constructs (e.g., public health literacy).

**Conclusion:**

Although there is a widely applied definition of distributed health literacy, our review revealed that the research space would benefit from the development of the concept, both theoretically for example via conceptual distinctions between distributed health literacy and other types of social support, and empirically for example through the development of a quantitative measurement instrument.

**Patient or Public Contribution:**

This paper is a systematic review and did not involve patients or the public.

## 1 INTRODUCTION

Health literacy has historically been defined as an observable set of individual skills that inform health actions. This individual focus is evident, for example, in the definition adopted by the World Health Organization in 1998—‘the personal, cognitive and social skills which determine the ability of individuals to gain access to, understand and use information to promote and maintain good health’,^1^ and in newer definitions such as from the International Union of Health Promotion, where health literacy is defined as the combination of personal competencies and situational resources needed for people to access, understand, appraise and use information and services to make decisions about health. It includes the capacity to communicate, assert and act upon these decisions.^2^ It is also clear from health literacy measurement instruments, which typically assess individual skills, such as skills in interpreting nutrition labels (e.g., the Newest Vital Sign),^3^ recognizing medical terms (e.g., the Rapid Estimatre of Adult Literacy in Medicine),^4^ and health‐related reading and numeracy skills (e.g., Test of Functional Health Literacy in Adults).^5^

However, more recently, the literature has begun to draw attention to the intersection between health literacy and the social context, acknowledging that other individuals, families and communities also play a role in one's health information acquisition, comprehension and decision‐making.^6^, ^7^, ^8^, ^9^ Most often, social context is modelled as a construct that impacts health but is *distinct from* health literacy. In a recent systematic review, for example, 23 of 34 identified studies represented social context in this way, measuring an *association* between health literacy and a social context variable.^6^ This included, for instance, measuring whether people with lower health literacy had more or less social support, social capital and social engagement compared to those with higher health literacy. Alternatively, a smaller number of studies (*n* = 6) positioned social skills (i.e., the ability to interact with and draw upon others for support) as a specific *type of* individual health literacy.^6^ This is also illustrated in the ‘Social support for health’ subscale of the Health Literacy Questionnaire.^10^

Other work still has recognized that an individual's health literacy skills are supplemented by those of others (including family, carers and health professionals), together contributing to an improvement in individual or collective health outcomes. Edwards et al.^7^ ‘distributed health literacy’ model, for example, argues that while individual health literacy may vary within a group, individuals can overcome personal deficits in health literacy skills by combining their efforts. In this way, distributed health literacy is a resource that may buffer the adverse impacts of low health literacy.^11^ Although previous research has provided a broad overview of the intersection between health literacy and the social context,^6^ to date there is no focused exploration of how *distributed health literacy* has been defined, conceptualized or assessed in the peer‐reviewed literature, and no attempt to synthesize the existing body of research. To progress this field, this study aims to explore: (1) definitions and conceptual models of distributed health literacy that are available from the peer‐reviewed literature; and (2) how distributed health literacy has been assessed in empirical research (including in quantitative and qualitative studies).

## 2 METHODS

### 2.1 Protocol and registration

This systematic review was registered on the Joanna Briggs Institute (JBI) Systematic Review Register and is reported in accordance with the Preferred Reporting Items for Systematic reviews and Meta‐Analyses 2020 statement.^12^

### 2.2 Eligibility criteria

For this review, we included articles published in peer‐reviewed journals that specifically:

1. Aimed to develop a new or refined definition or conceptual description of distributed health literacy based on theoretical or empirical data; or
2. Defined an analysis of distributed health literacy in the methods; or
3. Reported aspects or processes relating to distributed health literacy in the results.

We excluded other publication types (e.g., dissertations, books, conference abstracts) and articles that suggested exploring distributed health literacy as a future direction if this was not directly related to the aims, methods or results of the manuscript. To attain the widest range of studies, no limits were set for the language or date of publication.

### 2.3 Information sources and search strategy

The article search for this review was completed on 4 February 2021. The search strategy aimed to find published journal articles on the topic of distributed health literacy using a two‐step search strategy:

1. An initial search of MEDLINE, Embase, CINAHL, PsycInfo, Scopus, ERIC and Web of Science was undertaken. Keywords used were truncated versions of ‘literacy’ and ‘distributed’ (within five words' distance). Where controlled vocabulary terms (e.g., MeSH) were available they were applied. The full search strategies for all databases, including any limits used, are included in Appendix SA.
2. The reference lists of all eligible articles were checked for any additional relevant studies.

### 2.4 Study selection process

Two reviewers (D. G. and S. H. or D. M. M) independently screened all titles and abstracts using EPPI‐Reviewer prior to retrieving full texts, which were again independently screened by two reviewers for eligibility. Any disagreements between the reviewers were resolved through discussion with a third member of the research team (O. N. or D. M. M).

### 2.5 Data collection process and data items

Two reviewers (D. G. and S. H. or H. T. M. or D. M. M) independently extracted data from included papers using data extraction tools from the JBI. The data extracted included basic information about the study (author, year of publication, journal), study details (research questions, target groups, methods, settings, recruitment procedures, participant demographics, data analysis) and information related to distributed health literacy (definitions, conceptual models, instruments and approaches used to measure distributed health literacy, findings and authors' conclusions). For the purposes of this review, we did not seek to obtain or confirm data from study investigators.

### 2.6 Synthesis of results

In line with our study aims, we collated the definitions and conceptual models of distributed health literacy that were referred to in the included studies, and report on how health literacy has been measured in empirical research studies (including quantitative measurement tools and qualitative interview guides).

In addition, we also synthesized the results from all included qualitative studies (including systematic reviews of qualitative studies) and theoretical manuscripts using thematic synthesis, as described by Harden and Thomas.^13^ We took an inductive approach to find themes, which involved free line‐by‐line coding of the findings of primary studies and the organization of these ‘free codes’ into themes. Each stage was conducted by two authors (D. M. M. and D. G.), with discussion and input from the entire authorship team.

### 2.7 Quality appraisal

Full texts selected for retrieval were assessed for quality using standardized critical appraisal instruments from the JBI (https://joannabriggs.org/ebp/critical_appraisal_tools). Due to the heterogeneity of the study designs included, three JBI Critical Appraisal Tools were utilized to assess study quality. These appraisal tools included the Checklist for Qualitative Research^14^ (*n* = 6; for example, ‘Is there congruity between the research methodology and the methods used to collect data?’), the Checklist for Systematic Reviews and Research Synthesis^15^ (*n* = 3; e.g., ‘Were there methods to minimise errors in data extraction’) and the Checklist for Quasi‐Experimental Studies^16^ (*n* = 1; e.g., ‘Were outcomes measured in a reliable way’). Any disagreements between reviewers (D. G. and I. R. H) were resolved through discussion with a third member of the research team.

## 3 RESULTS

### 3.1 Study selection

**Figure 1:**
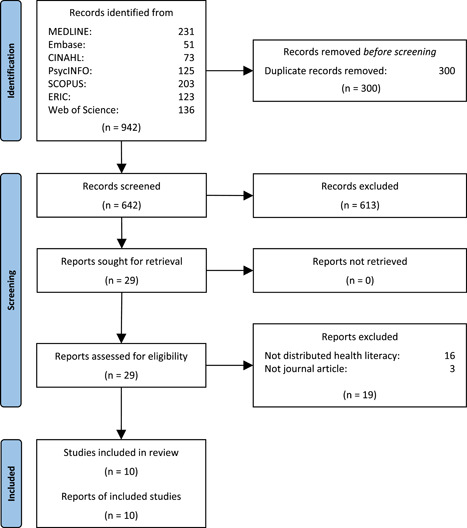
Flow diagram of study selection

Of the 642 studies screened, 10 were deemed to meet the inclusion criteria for this systematic review after full‐text screening (see Figure 1). The majority of included studies reported on qualitative research (*n* = 6). Other studies reported on quantitative research (secondary analysis of data; *n* = 1), or took the form of a systematic review (*n* = 1), conceptual analysis (*n* = 1) or ‘perspective’ article (*n* = 1).

### 3.2 Study characteristics

Table 1 provides summary details of the original research studies (qualitative and quantitative) included in this review. Qualitative studies included participants with a range of health conditions (e.g., type 2 diabetes, asthma, gestational diabetes, human immunodeficiency virus [HIV]) and were conducted in five countries, two of which were with ethnic minority groups.^7^, ^17^, ^18^, ^19^, ^20^, ^21^ Lorini and colleague's^22^ secondary analysis included quantitative data from Austria, Bulgaria, Greece, Poland and Spain.

**Table 1:** Summary details of included original research studies and approaches to assessing distributed health literacy

Author	Aims and/or research questions	Study population	Study location	Study type		Methods	Approaches to assessing distributed health literacy
				Qual	Quant		
Edwards et al. (2015)^7^	To examine the ‘distributed’ nature of health literacy and explore how participants with a range of long‐term conditions draw on people within their social network(s) for support with health literacy‐related tasks	People living with a long‐term health condition	United Kingdom	✓		Longitudinal qualitative methodology (including serial interviews)	Included interview questions that sought to identify ‘in what situations participants were supported by the health literacy, knowledge or skills of others (e.g., in searching for online information, making informed decisions and communicating with health professionals)’
Abreu et al. (2018a)^19^	How do patients with type 2 diabetes draw on their social network for support with identified health literacy‐related tasks?	People living with type 2 diabetes	Porto District, Northern Portugal	✓		Qualitative interviews	Included two topic questions to explore the role of ‘health mediators’ for people diagnosed with asthma and type two diabetes (Do you usually go accompanied to the medical visits (if yes, by whom)? If we ask you to choose someone to help you in a health‐related issue, who would you choose and why?). Provide a visual ‘map’ of health literacy mediators (e.g., partner; children) and practices (e.g., attends consultations; gives advice)
Abreu et al. (2018b)^20^	How do adults diagnosed with asthma draw on their social network for support with health literacy‐related tasks, mapping out health literacy mediators for each individual, and how they enable self‐management skills and knowledge about asthma?	Adults diagnosed with asthma	Porto District, Northern Portugal	✓		Qualitative interviews	As above
Dayyani et al. (2019)^17^	To explore how non‐Western ethnic minority pregnant women with gestational diabetes (GDM) in Denmark experience the hospital‐based information about GDM and how they integrate this information into their everyday life. The secondary aim was to investigate the role played by health literacy and distributed health literacy	Non‐Western ethnic minority pregnant women with gestational diabetes	Aarhus University Hospital, Denmark	✓		Qualitative interviews	Asked participants about their experience with different health professionals and whether and how they receive support from family and friends about how to live with gestational diabetes
McKinn et al. (2019)^21^	To examine the nature of maternal health literacy in Dien Bien Provence by exploring which formal and informal sources of health information ethnic minority women access and trust, and how women draw on these resources and their social and family networks to apply their understanding of health information and make health decisions	Ethnic minority women who were currently pregnant, or mothers or grandmothers of children younger than age 5 years	Dien BienProvence, Vietnam	✓		Focus groups	Asked three explicit questions about the role of family and community during their focus groups (How is your family involved with the baby? Does anyone give you advice about the pregnancy and having the baby? Are there things that your family/community expect you to do while you are pregnant?)
Uwamahoro et al. (2019)^18^	What health literacy‐related knowledge and skills do Young People Living with HIV (YPLHIV) in Malawi require to cope with life, take control of adverse social and environmental circumstances, and live healthy lives? How can the existing health literacy frameworks (functional, critical, interactive and distributed health literacy) be modified to incorporate the specific needs of YPLHIV in Malawi?	HIV‐positive young people (YPLWHIV) aged 18–35 years	Southern (Blantyre), Central (Lilongwe) and Northern (Mzuzu) regions, Malawi	✓		Focus group discussions and semi‐structured one‐to‐one interviews	Contextualized the definitions of four health literacy dimensions (including distributed health literacy) to ensure appropriateness to HIV in Malawian youth, and report that this initial conceptualization was used to develop the discussion and interview guides for their study
Lorini et al. (2020)^22^	Aim: To assess the role of health literacy as the country‐level ecological variable in predicting the health disparities among immigrants in different European Union countries. Research Question: Does the health literacy of a country influence the health disparities among immigrants?	Immigrants living in eight European countries	Austria, Bulgaria, Germany, Greece, Ireland, the Netherlands, Poland and Spain		✓	Secondary analysis of data	Country‐level health literacy data were obtained from the publicly available first European Health Literacy Survey reports. Individual‐level data on citizenship, perceived health status, body mass index, smoking habits, physical activity and attendance at breast and cervical cancer screening were extracted from the European Health Interview Survey of Eurostat. The country‐specific odds ratio (OR) for the association between the participants' citizenship and other individual health‐relevant characteristics was pooled into summary OR using random‐effects models. Meta‐regression was used to explore whether the health literacy of a country could explain part of the between‐countries heterogeneity. Health literacy was measured ecologically using the HLS‐EU‐Q47, with the authors including the average value and proportion of the country population with values of health literacy judged as ‘inadequate’ or ‘problematic or inadequate’ in their meta‐regression analysis

In addition to the primary studies, Gessler and colleagues^23^ conducted a systematic review of qualitative studies that explored the process of decision‐making and characterized how adolescents and young adults share healthcare information. Although the 14 eligible studies included in the review did not refer specifically to distributed health literacy, findings were synthesized using this conceptual frame. Bröder and colleagues conducted an iterative conceptual analysis of child and youth health literacy and offered a target‐group‐centred definition that embodied concepts of distributed health literacy.^24^, ^25^

### 3.3 Quality appraisal

Quality appraisal scores can be found in Tables S1–S3. Qualitative studies were rated as high quality overall, while the quantitative study and two studies assessed using the checklist for systematic reviews and research synthesis had lower quality ratings, mainly because key details of methodological rigour were not elucidated in the text (e.g., details about data extraction; critical appraisal).

### 3.4 Definitions and conceptual models of distributed health literacy available from the peer‐reviewed literature

#### 3.4.1 Definitions

In their seminal paper, Edwards and colleagues^7^ define distributed health literacy as ‘…the health literacy abilities, skills and practices of others that contribute to an individual's level of health literacy’. This definition was developed from a longitudinal qualitative interview and observation study of the development and practice of health literacy in people with long‐term health conditions, and built on previously published general literacy studies. For example, the manuscript references Wagner et al.,^26^ in the introduction, acknowledging their contribution in recognizing that ‘several individuals may each possess only some aspects of literacy, and by combining their efforts, they may function as more fully literate individuals’. Edwards and colleagues^7^ also refer to Baynham's^27^ ‘literacy mediators’ (i.e., people who make their literacy skills available to others, on a formal or informal basis, for them to accomplish specific literacy purposes) as well as Papen's^28^ application of this concept to the healthcare context.

Of the remaining studies included in this review, most drew on the work of Edwards et al.^7^ as a starting point, referring at least to their definition of distributed health literacy in the introductory manuscript text. A smaller number of studies continued to reference Wagner's distributed literacy either alone or in combination with Edward's definition when first defining the concept (e.g., Uwamahoro et al.).^18^

Uwamahoro and colleague's^18^ definition differs from that of Edwards in the focus on ‘the skills required to access social support which entails help to access, evaluate and understand information and make decisions regarding health from people in one's social network’. The authors note that, in an HIV context, this includes the ability to disclose one's HIV status and the ability and willingness of people in one's social network to offer the support needed.

Although Bröder et al.^25^ do not define distributed health literacy per se, they present a new ‘target‐group‐centred health literacy definition for children and young people in the results of their manuscript which positions health literacy as ‘a social and relational construct’. Drawing on the findings of their conceptual analysis, the authors acknowledge that children and young people's health literacy can be promoted by social structures in a variety of contexts and include ‘Social Health Literacy Assets’ as a conceptual dimension within their definition.^25^ This includes the social and cultural resources one can access via social support structures in the close social environment (family/peer/community context).

#### 3.4.2 Conceptual models

As well as offering a definition of distributed health literacy, Edwards et al.^7^ also provide a conceptual model known as the ‘Supported Health Literacy Pathway’. The model identifies intervention points at which people in a participant's social network influence that individual's health literacy through (1) shared health knowledge; (2) supported skills and practices; (3) supported actions; (4) coproduced informed options and (5) supported decisions.

Other work has included distributed health literacy as a component within larger models of health literacy. Uwamahoro and colleagues  for example, include distributed health literacy as a feature of both individual and system health literacy in their ‘contextual model’. Interestingly, distributed health literacy and public health literacy (i.e. the degree to which individuals and groups can obtain, process, understand, evaluate and act upon information needed to make public health decisions that benefit the community; Freedman et al.)^8^ are considered separate constructs in the model developed by Uwamahoro et al.^18^ This is inconsistent with other studies identified in our review that have used ‘public’ and ‘distributed’ health literacy interchangeably.^22^

### 3.5 How distributed health literacy has been assessed in empirical research

#### 3.5.1 Quantiative measurement

We did not identify any quantitative measures of distributed health literacy. However, Lorini et al.^22^ used data collected from pre‐existing population studies (European Health Literacy Survey Reports; European Health Interview Survey of Eurostat) to assess whether health literacy as the country‐level ecological variable predicts health disparities among immigrants in different European Union (EU) countries, including Austria, Bulgaria, Greece, Poland and Spain. Here, health literacy was measured ecologically using the HLS‐EU‐Q47, with the authors including the average value and proportion of the country population with values of health literacy judged as ‘inadequate’ or ‘problematic or inadequate’ in their meta‐regression analysis.^22^ Although this approach is arguably less aligned with existing definitions of distributed health literacy, the study was included as the authors note the aim was to advance ‘understanding of the role of distributed health literacy’.

#### 3.5.2 Qualitative measurement

Of the qualitative studies identified, Edwards and colleagues^7^ originally included interview questions that sought to identify ‘in what situations participants were supported by the health literacy, knowledge or skills of others (e.g., in searching for online information, making informed decisions and communicating with health professionals)’. Since then, the number, type and depth of interview/focus group questions related to distributed health literacy have varied. See Table 1 for examples.

#### 3.5.3 Systematic reviews and conceptual analyses

The systematic reviews and conceptual analyses that we identified rarely included methods or search strategies specific to distributed health literacy. Rather, they adopted a broad approach to searching the health literacy literature, framing their results and/or forming conclusions and definitions of distributed health literacy from the analysis of their findings (see Table 2).

**Table 2:** Summary details of included systematic reviews and conceptual analyses

Author	Study type	Aims and/or research questions	Data sources and search terms	Analysis and synthesis	How was distributed health literacy included?
Gessler et al. (2019)^23^	Systematic review	To identify and synthesize qualitative studies that have addressed the interactional process facilitating empowerment and participation in shared decision‐making in adolescents and young adults and their families	Searched Embase, MEDLINE, PsycInfo and CINAHL. Search strategy combined the concept of health literacy with the involvement of family members. As such, health literacy was captured using a broad range of search terms, including shared decision‐making, patient participation patient involvement, health literacy, patient communication, empowerment and patient engagement. Family involvement in patient care was captured by search terms, including parent, triad, carer, caregiver, family, sibling and partner	Data were analysed using the Framework method. During the early data analysis, authors identified that a subset of themes aligned with the Supported Health Literacy Pathway Model, so a hybrid process of inductive and deductive coding was used to analyse data	In the results, a central theme was: ‘Distribution of health literacy skills among AYA‐family–clinician triads’, which included the following subthemes: 1. Shared health knowledge 2. Supported skills and practices 3. Supported action 4. Coproduced informed options 5. Supported decisions
Bröder et al.^24^ (2020)	Literature review	To discuss children's and young people's health literacy by elaborating and exploring childhood and youth as life phases with unique characteristics from multidisciplinary perspectives	Specific data sources and search terms not specified—‘We studied literature from childhood studies, educational and sociological research to identify and explore unique particularities of children and young people that are of relevance for health literacy research and practice’	Adapted the ‘D’ framework used by Rothman et al.^29^ (2009) : (1) Disease patterns and health perspectives, (2) demographic patterns: contextual factors and inequalities, (3) developmental change: socialization and life course perspective, (4) dependency: power structures and intergenerational relationships, and (5) democracy: active citizenship and participation	Distributed health literacy was included in the dimension: ‘Dependency: power structures and intergenerational relationships’ through the recognition that children's and young people's ‘agency can be regarded as being determined by the opportunities presented within the different social contexts, demographic and socioeconomic circumstances, as well as the distributed resources’
Bröder et al. (2020) ^25^	Conceptual analysis	To analyse, examine and reflect upon prominent health literacy understandings in childhood and youth	Applied an iterative process to ‘search for and analyse relevant, multidisciplinary literature from childhood studies, educational science, and sociology’. Also ‘drew on the results of a systematic review of available conceptual understandings of health literacy for children and young people’	Analysed the identified body of literature to identify attributes and components of health literacy. The identified attributes and components were deconstructed and categorized by clarifying their characteristics, their assumptions, and their relation towards each other. Results were grouped and synthesized through an iterative process facilitated by reflective and analytical discussions within the research team	Results acknowledged that identified studies addressed the health literacy of persons close to the child, such as caregivers, mothers, parents and teachers, and noted that ‘researchers have proposed that child and adolescent health literacy should be regarded as the product of both individual health literacy skills and the skills or resources available in the proximal social context—namely, the adults, peers or institutions that young people trust. Among others, this is referred to as “collective” or “distributed” health literacy’. In proposing a target‐group‐centred health literacy definition for children and young people, authors note that the relatedness and contextual embeddedness of health literacy is placed at the core of this definition by recognizing individual and distributed resources within given structures. Health literacy is considered as being socially embedded and distributed on individual, family and social levels

### 3.6 Synthesis of findings related to distributed health literacy

Through our thematic synthesis, we identified two themes from the data:

1. From social support to distributed skills and practices
2. Limited network density and non‐supportive roles

#### 3.6.1 Theme 1: From social support to distributed skills and practices

All studies identified health literacy as a distributed attribute. However, the scope of what was included as distributed health literacy varied. While some focused on distributed skills and practices, others included more general forms of social support.

*Distributed skills and practices*: In their results, four studies specifically describe a range of health literacy skills and practices that were distributed around an individual by members of their social network. Abreu et al.,^19^ for example, found that a core network of health mediators provided most health literacy competencies for their participants (e.g., preventing exposure to certain environments or preventing symptoms, helping in moments of crisis and intake of quick‐relief medication, understanding and obtaining health information, seeking online information). In their analysis of qualitative focus group data, McKinn et al.^21^ found that themes aligned with the conceptualization of health literacy by Edwards et al.^7^ and therein presented their results under four subsections corresponding to the areas of distributed health literacy described by Edwards et al. In the context of pregnancy and parenthood in the Dien Bien Provence, Vietnam, ethnic minority women drew upon family and social networks to share knowledge and understanding, assess and evaluate information, communicate with health professionals and support decision‐making.

Using a hybrid process of inductive and deductive coding, Gessler et al.^23^ organized their qualitative synthesis according to the five steps in Edwards' Supported Health Literacy Pathway Model, finding support across all the reviewed studies for the application of this model into the Adolescent and Young Adult (AYA) cancer setting. The authors concluded that AYAs develop their health literacy in partnership with their families, who support and share knowledge about the health conditionand skills and practices associated with managing the condition, as well as being actively involved in discussions with clinicians, producing informed options and making informed decisions.

*Social support*: The remaining studies, however, mainly focused on family and social networks as sources of ‘social support’, rather than providing or supplementing a range of health literacy *skills*. Uwamahoro et al.,^18^ for example, reported that their participants identified meeting other young people living with HIV (e.g., through teen clubs at clinics and community support groups) as an important determinant of health and a coping strategy. Their qualitative analysis of distributed health literacy focused on social and community networks as assisting young people living with HIV to ‘break the isolation by enabling them to meet others in the same situation’. While the authors did note that family was also a source of information as well as material and emotional support, this was offered as a single sentence in a much larger description of social support and self‐stigma in the ‘distributed health literacy’ theme of their qualitative analysis. Dayyani et al.^17^ also found that family could be supportive in the context of women with gestational diabetes; the mothers of participants were often highlighted as ‘emotional supporters’, sharing their own experiences with diabetes. Although one participant described how her partner found health information related to gestational diabetes on her behalf, the qualitative analysis did not elucidate or explore any other distributed skills and practices. In the discussion of their manuscript, however, Dayyani et al.^17^ acknowledged that ‘to have access to health literacy of friends and relations, non‐Western ethnic minority pregnant women with GDM need to have social networks supporting them’.

#### 3.6.2 Theme 2: Limited network density and non‐supportive roles

A number of studies also noted the absence of support structures in their discussion of health literacy. Two studies reported that there were barriers to distributed health literacy for some of their participants (e.g., HIV‐related stigma) and/or that some people did not have many or any supporters in their health journey.^17^, ^18^ This was reinforced by Abreu et al.,^19^ when they described two identities with different distributed health literacy ‘profiles’. One profile was exemplified by what they termed ‘a narrative of minimization’ whereby individuals reported a dense network of health literacy mediators. These participants claimed low impact of asthma on their lives and daily routines, easy control of symptoms and avoidance of major crises. They could rely on their primary care physician for instrumental support and on close family members with asthma to provide emotional and pragmatic support (e.g., related to medication use) and alert them to situations that might trigger an asthma attack. The second profile, however, was referred by the authors as ‘one of disruption’, enacted by interviewees who relied on a restricted network of core health mediators made up of formal sources of health services (clinical interaction or online) used mainly to provide informational support. They described episodes of crisis as highly disruptive, participants' difficulties in controlling crises and their feelings of stigma. These participants tended to hide asthma and to look for alternative and complementary solutions to control anxiety, demonstrating a reactive approach to asthma management.^20^

Other studies have identified the disruptive or non‐supportive role that other individuals may play in the acquisition and use of health literacy skills. McKinn et al.,^21^ for example, reported that social networks and collective decision‐making at times had a negative effect on health behaviours for ethnic minority women in Dien Bien Provence, Vietnam. This is exemplified by the experience of a participant who mentioned that exclusive breastfeeding could be interrupted, and weaning commenced earlier than advised based on the preferences of their parents‐in‐law. Uwamahoro et al.^18^ also noted that by providing special treatment to the HIV‐positive client (and supporting distributed health literacy) at times exposed them to stigma in the process. In their conceptual analysis, Bröder et al.^24^, ^25^ also acknowledge that health literacy can be hindered by social structures, power relationships, societal demands and layers of autonomy.

## 4 DISCUSSION

This systematic review aimed to explore the way in which distributed health literacy has been defined, conceptualized and assessed in the peer‐reviewed literature to date, and to synthesize existing literature in this field. We identified few studies reporting on the concept to date. Of the included studies, Edwards' definition of distributed health literacy, which emphasizes the health literacy abilities, skills and practices of others that contribute to an individual's level of health literacy, was widely applied across different clinical and geographical settings. Four qualitative studies described in detail the way in which individuals draw upon family and social networks to share knowledge and understanding, assess and evaluate information, communicate with health professionals and support decision‐making. Others more simply reported family and friends as sources of information as well as material and emotional support. Together with discrepancies in the positioning of distributed health literacy in relation to other concepts (e.g., public health literacy), our findings raise important questions about what is meant by distributed health literacy and what the edges of that construct are. We also identified an array of qualitative assessment approaches, but no quantitative instruments to directly measure distributed health literacy.

The findings of this review reinforce the utility of approaching health literacy as a distributed attribute and not exclusively individual. However, variation in the way in which ‘distributed health literacy' was assessed and described across studies suggests that this emerging field of research would benefit from additional conceptual clarity. Particularly important is the need to differentiate between the support that supplements or compensates for individual health literacy skills and other types of support that one might receive on their health journey (e.g., general social/emotional support). We suggest that the historical focus on *skills* is key to this definitional challenge, with a specific emphasis on skills that supplement or bolster the health literacy of the patient. For example, a carer finding health information on someone's behalf would represent distributed health literacy, while having a neighbour with whom to confide in about challenges of living with a health condition would not. Moving forward with more defined parameters about what constitutes distributed health literacy and what does not will expectedly help to ensure that the concept offers a unique contribution—both theoretically and practically—over and above existing concepts (e.g., social support; social capital), and avoids previous critiques of health literacy as ‘new wine in old bottles’.^30^ It may also support more systematic literature searching and help to replace the broad and varied approaches used to date. More work is also required to delineate between the concepts of public health literacy, community health literacy and distributed health literacy; a concept analysis, for example, may be an appropriate method to achieve this.

From a measurement perspective, this review highlights the need for quantitative measures of distributed health literacy. While qualitative approaches allow for deep exploration of distributed health literacy as a concept, a quantitative tool would enable quicker assessment, help to validate the construct and underlying theory, and facilitate the measurement of change in distributed health literacy over time and/or with the implementation of health literacy interventions. A concept mapping approach may be particularly useful in developing such a measure,^31^ particularly as measures will need to carefully delineate between general social support and support that compensates for individual health literacy skills. Measures should also seek to capture the distribution of responsibilities among those involved in shared health literacy practices. This could entail looking at (a) the sum of health literacy resources available within one's proximal social context and community, and (b) how these resources are then used. In interpersonal psychotherapy, an ‘interpersonal inventory’ is used to identify people within a patient's network using concentric circles (closest on the inside).^32^ We feel that something similar could also be applied in the measurement of distributed health literacy. Given the findings of this review, there may also be utility in trying to capture potentially nonsupportive networks, and specifically analysing distributed health literacy in the social context of children and adolescents.

Finally, our review makes evident that much work in the area of distributed health literacy has occurred with children and young adults, in developing countries and/or with ethnic‐minority groups. Given the large body of research that exists regarding culture and individualist/collectivist orientations,^33^ and the unique social context of children, it is unsurprising that researchers have chosen to explore social networks and distributed health literacy in these groups. Moving forward, it would be useful to explore, compare and contrast the validity of these similar constructs among different groups.

## 5 STRENGTHS AND LIMITATIONS

We adopted very focused search terms in this review, purposefully including variants of ‘distributed’ as a key term. In this way, we would have excluded a number of studies that explore the intersection between health literacy and the social context more generally without referring specifically to the concept of ‘distributed health literacy’, and biased results towards studies published after 2015. However, this matches our study aims and avoids duplication of research which has already been done. Our findings are also limited by the small number of identified studies, although they do represent five diverse countries across the globe and participants with various health conditions.

Blinding and independent assessments of articles for inclusion and risk of bias represent an important strength. We searched seven databases for relevant studies and checked reference lists to supplement our searching, although we acknowledge that not all potential databases were searched, which may have resulted in some missed articles.

## 6 CONCLUSION

Although there is a rather widely applied definition of distributed health literacy, the research space would benefit from additional conceptual clarity. This includes the development of the concept, both theoretically for example via conceptual distinctions between distributed health literacy and other health literacy concepts, as well as other types of social support, and empirically for example, through the development of one or more quantitative measurement instruments.

## CONFLICT OF INTERESTS

The authors declare that there is no conflict of interests.

## AUTHOR CONTRIBUTIONS

*Concept and design*: Danielle M. Muscat, Helle T. Maindal, Ole Norgaard, Julie Ayre, Danielle Gessler. *Acquisition, analysis or interpretation of data*: All authors. *Drafting of the manuscript*: Danielle M. Muscat, Julie Ayre and Danielle Gessler. Critical revision of the manuscript for important intellectual content: All authors.

## Supporting information

Supporting information.

Supporting information.

## Data Availability

All data relevant to the study are included in the article or uploaded as Supporting Information.

## References

[1] Nutbeam D . Health literacy as a public health goal: a challenge for contemporary health education and communication strategies into the 21st century. Health Promot Int. 2000;15(3):259‐267. 10.1093/heapro/15.3.259

[2] Bröder J , Chang P , Kickbusch I , et al. IUHPE position statement on health literacy: a practical vision for a health literate world. Glob Health Promot. 2018;25(4):79‐88. 10.1177/1757975918814421

[3] Weiss BD , Mays MZ , Martz W , et al. Quick assessment of literacy in primary care: the newest vital sign. Ann Fam Med. 2005;3(6):514‐522. 10.1370/afm.405 16338915 PMC1466931

[4] Davis TC , Long SW , Jackson RH , et al. Rapid estimate of adult literacy in medicine: a shortened screening instrument. Fam Med. 1993;25(6):391‐395. 8349060

[5] Parker RM , Baker DW , Williams MV , Nurss JR . The test of functional health literacy in adults. J Gen Intern Med. 1995;10(10):537‐541. 10.1007/BF02640361 8576769

[6] Sentell T , Pitt R , Buchthal OV . Health literacy in a social context: review of quantitative evidence. Health Lit Res Pract. 2017;1(2):e41‐e70. 10.3928/24748307-20170427-01 31294251 PMC6607851

[7] Edwards M , Wood F , Davies M , Edwards A . ‘Distributed health literacy’: longitudinal qualitative analysis of the roles of health literacy mediators and social networks of people living with a long‐term health condition. Health Expect. 2015;18(5):1180‐1193. 10.1111/hex.12093 23773311 PMC5060848

[8] Freedman DA , Bess KD , Tucker HA , Boyd DL , Tuchman AM , Wallston KA . Public health literacy defined. Am J Prev Med. 2009;36(5):446‐451. 10.1016/j.amepre.2009.02.001 19362698

[9] Kendir C , Breton E . Health literacy: from a property of individuals to one of communities. Int J Environ Res Public Health. 2020;17(5):1601. 10.3390/ijerph17051601 PMC7084319 32131441

[10] Osborne RH , Batterham RW , Elsworth GR , Hawkins M , Buchbinder R . The grounded psychometric development and initial validation of the Health Literacy Questionnaire (HLQ). BMC Public Health. 2013;13(1):658. 10.1186/1471-2458-13-658 23855504 PMC3718659

[11] Lee S‐YD , Arozullah AM , Cho YI . Health literacy, social support, and health: a research agenda. Soc Sci Med. 2004;58(7):1309‐1321. 10.1016/S0277-9536(03)00329-0 14759678

[12] Page MJ , McKenzie JE , Bossuyt PM , et al. The PRISMA 2020 statement: an updated guideline for reporting systematic reviews. J Clin Epidemiol. 2021;134:178‐189. 10.1016/j.jclinepi.2021.03.001 33789819

[13] Harden A , Thomas J . Methods for the thematic synthesis of qualitative research in systematic reviews. BMC Med Res Methodol. 2008;8(1):45. 10.1186/1471-2288-8-45 18616818 PMC2478656

[14] Lockwood C , Munn Z , Porritt K . Qualitative research synthesis: methodological guidance for systematic reviewers utilizing meta‐aggregation. Int J Evid Based Healthc. 2015;13(3):179‐187. 10.1097/XEB.0000000000000062 26262565

[15] Aromataris E , Fernandez R , Godfrey CM , Holly C , Khalil H , Tungpunkom P . Summarizing systematic reviews: methodological development, conduct and reporting of an umbrella review approach. Int J Evid Based Healthc. 2015;13(3):132‐140. 10.1097/XEB.0000000000000055 26360830

[16] Tufanaru C , Munn Z , Aromataris E , Campbell J , Hopp L . Chapter 3: Systematic reviews of effectiveness. In: Aromataris E , Munn Z , eds. Joanna Briggs Institute Reviewer's Manual, The Joanna Briggs Institute; 2017. Available from https://reviewersmanual.joannabriggs.org/

[17] Dayyani I , Terkildsen Maindal H , Rowlands G , Lou S . A qualitative study about the experiences of ethnic minority pregnant women with gestational diabetes. Scand J Caring Sci. 2019;33(3):621‐631. 10.1111/scs.12655 30653703

[18] Uwamahoro NS , Ngwira B , Vinther‐Jensen K , Rowlands G . Health literacy among Malawian HIV‐positive youth: a qualitative needs assessment and conceptualization. Health Promot Int. 2019;35(5):1137‐1149. 10.1093/heapro/daz107 31691797

[19] Abreu L , Nunes JA , Taylor P , Silva S . Distributed health literacy among people living with type 2 diabetes in Portugal: defining levels of awareness and support. Health Soc Care Community. 2018;26(1):90‐101. 10.1111/hsc.12465 28643399

[20] Abreu L , Nunes JA , Taylor P , Silva S . The role of distributed health literacy in asthma integrated care: a public medical context from Portugal. Int J Integr Care. 2018;18(2):18. 10.5334/ijic.3301 30127702 PMC6095081

[21] McKinn S , Linh DT , Foster K , McCaffery K . Distributed health literacy in the maternal health context in Vietnam. Health Lit Res Pract. 2019;3(1):e31‐e42. 10.3928/24748307-20190102-01 31294305 PMC6608917

[22] Lorini C , Caini S , Ierardi F , Bachini L , Gemmi F , Bonaccorsi G . Health literacy as a shared capacity: does the health literacy of a country influence the health disparities among immigrants? Int J Environ Res Public Health. 2020;17(4):1149. 10.3390/ijerph17041149 PMC7068321 32059496

[23] Gessler D , Juraskova I , Sansom‐Daly UM , Shepherd HL , Patterson P , Muscat DM . Clinician‐patient‐family decision‐making and health literacy in adolescents and young adults with cancer and their families: a systematic review of qualitative studies. Psycho‐oncology. 2019;28(7):1408‐1419. 10.1002/pon.5110 31108019

[24] Bröder J , Okan O , Bauer U , Schlupp S , Pinheiro P . Advancing perspectives on health literacy in childhood and youth. Health Promot Int. 2020;35(3):575‐585. 10.1093/heapro/daz041 31143943

[25] Bröder J , Okan O , Bollweg TM , Bruland D , Pinheiro P , Bauer U . Child and youth health literacy: a conceptual analysis and proposed target‐group‐centred definition. Int J Environ Res Public Health. 2019;16(18):3417. 10.3390/ijerph16183417 PMC6765952 31540040

[26] Wagner DA , Messick BM Spratt JE. Studying literacy in Morocco. In: Schiefellin BB , Gilmore P , eds. The Acquisition of Literacy: Ethnographic Perspectives. Ablex; 1986:233–260.

[27] Baynham M . Literacy practices: investigating literacy in social contexts. Longman; 1995:

[28] Papen U . Literacy, learning and health–a social practices view of health literacy. Literacy & Numeracy Studies. 2009;16:19–34.

[29] Rothman RL , Yin HS , Mulvaney S , Co JPT , Homer C , Lannon C . Health literacy and quality: focus on chronic illness care and patient safety. Pediatrics. 2009;124:S315–S326. 19861486 10.1542/peds.2009-1163H

[30] Tones K . Health literacy: new wine in old bottles? Health Educ Res. 2002;17(3):287‐290. 10.1093/her/17.3.287 12120844

[31] Rosas SR , Ridings JW . The use of concept mapping in measurement development and evaluation: Application and future directions. Eval Program Plann. 2017;60:265‐276. 10.1016/j.evalprogplan.2016.08.016 27601290

[32] Fishman DB , Messer SB , Edwards DJA , et al. Case Studies within Psychotherapy Trials: Integrating Qualitative and Quantitative Methods. Oxford University Press, Incorporated; 2017.

[33] Hofstede G . Culture's Consequences: Comparing Values, Behaviors, Institutions, and Organizations Across Nations. 2nd ed. Sage Publications; 2001.

