# Supplementary material for: Seeking a deeper understanding of ‘distributed health literacy’: A systematic review
Source: Health Expect. 2022 Feb 18;25(3):856–68. doi: 10.1111/hex.13450 (PMC9122402; doi:10.1111/hex.13450)
Supplement: Supplementary file 1 — Supporting information. [file HEX-25--s002.docx]

**Appendix A: Search strategies**

**MEDLINE (Ovid)**

1 Health Literacy/ 6,315

2 distribut$.ti,ab,kf,kw. 1,120,206

3 1 and 2 178

4 (distribut$ adj5 literac$).ti,ab,kf,kw. 77

5 3 or 4 **231**

**Embase (Ovid)**

1 health literacy/ 12,760

2 distribut$.ti,ab,kw. 1,326,496

3 1 and 2 385

4 (distribut$ adj5 literac$).ti,ab,kw. 76

5 3 or 4 417

6 limit 5 to exclude medline journals **51**

**CINAHL (EBSCOhost)**

S1 (MH "Health Literacy") 3,409

S2 (TI distribut* OR AB distribut* OR SU distribut*) 39,799

S3 S1 AND S2 59

S4 TI (distribut* N5 literac*) 2

S5 AB (distribut* N5 literac*) 21

S6 SU (distribut* N5 literac*) 0

S7 S4 OR S5 OR S6 22

S8 S3 OR S7 **73**

*Limit: Exclude MEDLINE records*

**PsycInfo (Ovid)**

1 health literacy/ 3,201

2 distribut$.ti,ab,id. 120,944

3 1 and 2 80

4 (distribut$ adj5 literac$).ti,ab,id. 57

5 3 or 4 **125**

**Scopus**

TITLE-ABS-KEY(distribut* W/5 literac*) **203**

**ERIC (EBSCOhost)**

S1 TI (distribut* N5 literac*) 4

S2 AB (distribut* N5 literac*) 120

S3 S1 OR S2 **123**

**Web of Science**

TS=(distribut* NEAR/5 literac*) **136**
