# Supplementary material for: Seeking a deeper understanding of ‘distributed health literacy’: A systematic review
Source: Health Expect. 2022 Feb 18;25(3):856–68. doi: 10.1111/hex.13450 (PMC9122402; doi:10.1111/hex.13450)
Supplement: Supplementary file 2 — Supporting information. [file HEX-25--s001.docx]

**Supplementary File 1 – Quality Appraisal Results**

| **Supplementary Table 1.**  Quality Appraisal of Qualitative Studies using the Checklist for Qualitative Research (Lockwood, Munn, & Porritt, 2015)) | | | | | | | | | | |
| --- | --- | --- | --- | --- | --- | --- | --- | --- | --- | --- |
| **Studies** | **Quality Appraisal Item** | | | | | | | | | |
|  | 1. Is there congruity between the stated philosophical perspective and the research methodology? | 2. Is there congruity between the research methodology and the research question or objectives? | 3. Is there congruity between the research methodology and the methods used to collect data? | 4. Is there congruity between the research methodology and the representation and analysis of data? | 5. Is there congruity between the research methodology and the interpretation of results? | 6. Is there a statement locating the researcher culturally or theoretically? | 7. Is the influence of the researcher on the research, and vice- versa, addressed? | 8. Are participants, and their voices, adequately represented? | 9. Is the research ethical according to current criteria or, for recent studies, and is there evidence of ethical approval by an appropriate body? | 10. Do the conclusions drawn in the research report flow from the analysis, or interpretation, of the data? |
| Abreu et al,. 2018a | Unclear | Yes | Yes | Yes | Yes | No | No | Yes | Yes | Yes |
| Abreu et al,. 2018b | Unclear | Yes | Yes | Yes | Yes | No | No | Yes | Yes | Yes |
| Edwards et al., 2015 | Unclear | Yes | Yes | Yes | Yes | No | No | Yes | Yes | Yes |
| McKinn et al., 2019 | Yes | Yes | Yes | Yes | Yes | Yes | Yes | Yes | Yes | Yes |
| Dayyani et al., 2019 | Yes | Yes | Yes | Yes | Yes | Unclear | Yes | Yes | N/A | Yes |
| Uwamahoro et al., 2019 | Unclear | Yes | Yes | Yes | Yes | Yes | Yes | Yes | Yes | Yes |

| **Supplementary Table 2.**  Quality Appraisal of Systematic Reviews and Research Syntheses using the Checklist for Systematic Reviews and Research Synthesis (Aromataris et al., 2015) | | | | | | | | | | | |  |
| --- | --- | --- | --- | --- | --- | --- | --- | --- | --- | --- | --- | --- |
| **Studies** | **Quality Appraisal Item** | | | | | | | | | | |  |
|  | 1. Is the review question clearly and explicitly stated? | 2. Were the inclusion criteria appropriate for the review question? | 3. Was the search strategy appropriate? | 4. Were the sources and resources used to search for studies adequate? | 5. Were the criteria for appraising studies appropriate? | 6. Was critical appraisal conducted by two or more reviewers independently? | 7. Were there methods to minimize errors in data extraction? | 8. Were the methods used to combine studies appropriate? | 9. Was the likelihood of publication bias assessed? | 10. Were recommendations for policy and/or practice supported by the reported data? | 11. Were the specific directives for new research appropriate? | |
| Bröder et al., 2020 | Yes | N/A | N/A | Unclear | N/A | N/A | Unclear | Yes | No | Yes | Yes | |
| Bröder et al., 2019 | Yes | N/A | N/A | Yes | Unclear | N/A | Unclear | Yes | N/A | Yes | Yes | |
| Gessler et al., 2019 | Yes | Yes | Yes | Yes | Yes | Yes | Unclear | Yes | N/A | Yes | Yes | |

| **Supplementary Table 3.**  Quality Appraisal of Quasi-Experimental Studies using the Checklist for Quasi-Experimental Studies (Tufanaru, Munn, Aromataris, Campbell, & Hopp, 2020) | | | | | | | | | |
| --- | --- | --- | --- | --- | --- | --- | --- | --- | --- |
| **Studies** | **Quality Appraisal Item** | | | | | | | | |
|  | 1. Is it clear in the study what is the ‘cause’ and what is the ‘effect’? | 2. Were the participants included in any comparisons similar? | 3. Were the participants included in any comparisons receiving similar treatment/care, other than the exposure or intervention of interest? | 4. Was there a control group? | 5. Were there multiple measurements of the outcome both pre and post the intervention/exposure? | 6. Was follow up complete and if not, were differences between groups in terms of their follow up adequately described and analyzed? | 7. Were the outcomes of participants included in any comparisons measured in the same way? | 8. Were outcomes measured in a reliable way? | 9. Was appropriate statistical analysis used? |
| Lorini et al., 2020 | Yes | No | Yes | No | N/A | N/A | Yes | Yes | Yes |
